# Supplementary material for: Context-dependent neural preparation for information relevance vs. probability
Source: Imaging Neurosci (Camb). 2024 Oct 4;2:imag-2-00302. doi: 10.1162/imag_a_00302 (PMC12290532; doi:10.1162/imag_a_00302)
Supplement: Supplementary Material [file imag_a_00302-supp.pdf]

Supplementary Materials

Supplementary Table 4.1. Behavioral accuracy results.

Conditions	F	p	η^2_p
Block	0.311	0.580	0.007
Cueing	2.231	0.142	0.047
Category	13.024	< .001	0.224
Block ✻ Cueing	1.977	0.167	0.042
Block ✻ Category	24.112	< .001	0.349
Cueing ✻ Category	6.766	0.013	0.131
Block ✻ Cueing ✻ Category	6.717	0.013	0.130

Supplementary Table 4.2. Reaction Time results.

Conditions	F	p	η^2_p
Block	1.475	0.231	0.032
Cueing	9.451	0.004	0.174
Category	1.679	0.202	0.036
Block ✻ Cueing	1.291	0.262	0.028
Block ✻ Category	1.417	0.240	0.031
Cueing ✻ Category	0.679	0.414	0.015
Block ✻ Cueing ✻ Category	5.384	0.025	0.107

Supplementary Table 3. Univariate contrast results

Contrast	Region	Coordinates	Cluster size (k)	Z	p	T(peak)
Cue Att.> Cue Exp.	V2	8, -80, -12	216	3.62	0.04	3.94
Cue Exp.> Cue Att.	rpCC	2, -16, 40	367	3.9	0.004	4.3
	lpCC	-4, -42, -28	210	3.84	0.044	4.22

Notes: Region labeling based on the Julich-Brain atlas (Amunts, 2020). P-values are cluster values corrected for multiple comparisons. V2 = Secondary visual area, rpCC = right posterior Cingulate Cortex, lpCC = left posterior Cingulate Cortex. Att. = attention, Exp. = expectation.

Supplementary Table 4. Decoding accuracy results.

Contrast	Region	Coordinates	Cluster size (k)	Z	p	Decoding (peak)
Cue Att. vs. Exp.	SMA	-6, -20, 50	27841	5.46	<0.001	60.34%
	LiG	-6, -76, -6	2302	3.86	<0.001	57,69%
Target Att. vs. Exp.	PRG	34, -18, 52	54668	6.40	<0.001	62.21%
	STC	-48, -40, 2	1595	4.93	0.002	56,44%
Cue category decoding – Att.	lITG	-42, -58, -6	1564	5.73	0.001	59.91%
	rITG	42, -58, -10	1439	4.84	0.001	57.31%
Cue category decoding – Exp.	LiG	-20, -84, -8	2761	4.4	<0.001	57.46%

Note: Decoding accuracy (balanced accuracy) shows the accuracy value in the peak voxel. SMA = Supplementary motor area, LiG = lingual gyrus; PRG = Precentral Gyrus, STC = Superior Temporal Cortex, ITG = inferior temporal cortex, l and r = left and right.

Supplementary Table 5. Cross-decoding results

Contrast	Region	Coordinates	Cluster size (k)	Z	p	Decoding (peak)
Cue category between blocks	lFG	-42, -50, -22	756	4.01	0.026	54.13%
Cue-Target category – Train Cue - Att.	lITG	-48, -52, -8	1246	5.29	0.002	57.13%
	lFO	-52, 6, 20	594	4.17	0.03	54.17%
Cue-Target category – Train Cue- Exp.	lIOG	-38, -62, -2	8235	6.64	<0.001	57.12%
Cue-Target category – Exp. – Att.	V1	18, -86, 0	2544	4.59	<0.001	T = 5.26
Cue-Target category – Bidirectional - Att	lITG	-48, -52, 8	1291	5.3	0.002	57.23%
	lFO	-50, 4, 20	595	4.1	0.033	54.05%
Cue-Target category – Bidirectional - Exp	lIOG	-38, -62,2	8602	6.59	<0.001	57.2%
Cue category across different cues - Att	rFG	36, -60, 8	1248	4.93	0.002	55.36%
	S1	-40, -22, 44	1199	4.07	0.002	56.3%

IFG = Inferior Frontal Gyrus, lITG = left Inferior Temporal Gyrus, lFO = left Frontal Operculum, lIOG = left inferior occipital gyrus, lFG = left Fusiform Gyrus. rFG = right Fusiform Gyrus. Att. = attention, Exp. = expectation. S1 = Primary Somatosensory Cortex.

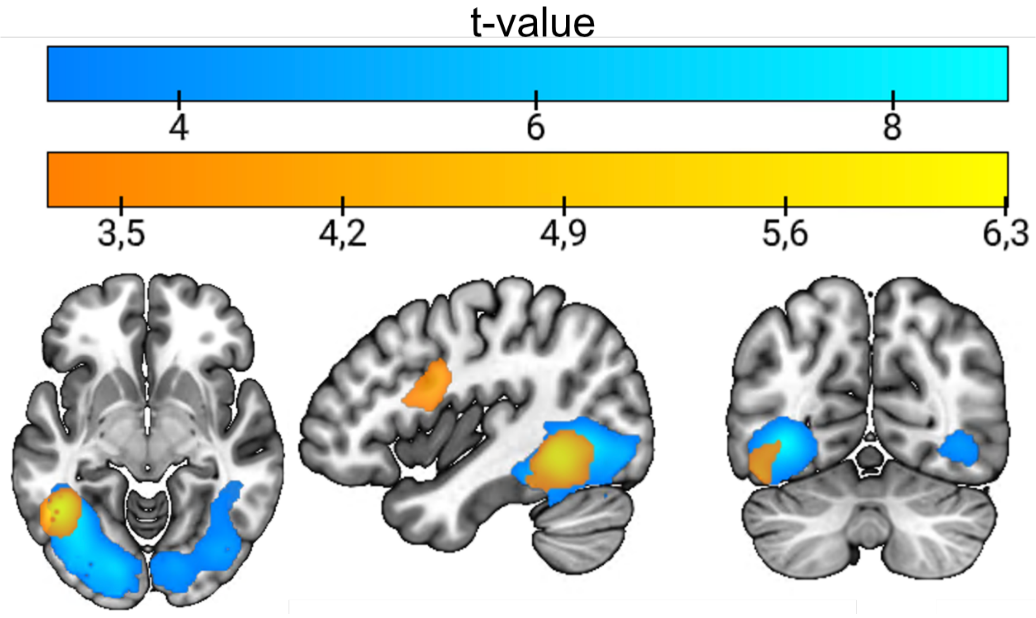

Supplementary Figure 1. Cue-Target Cross-decoding results, average of training and testing in the two two directions (cue-target and target-cue). Attention results are shown in yellow, and expectation in blue.

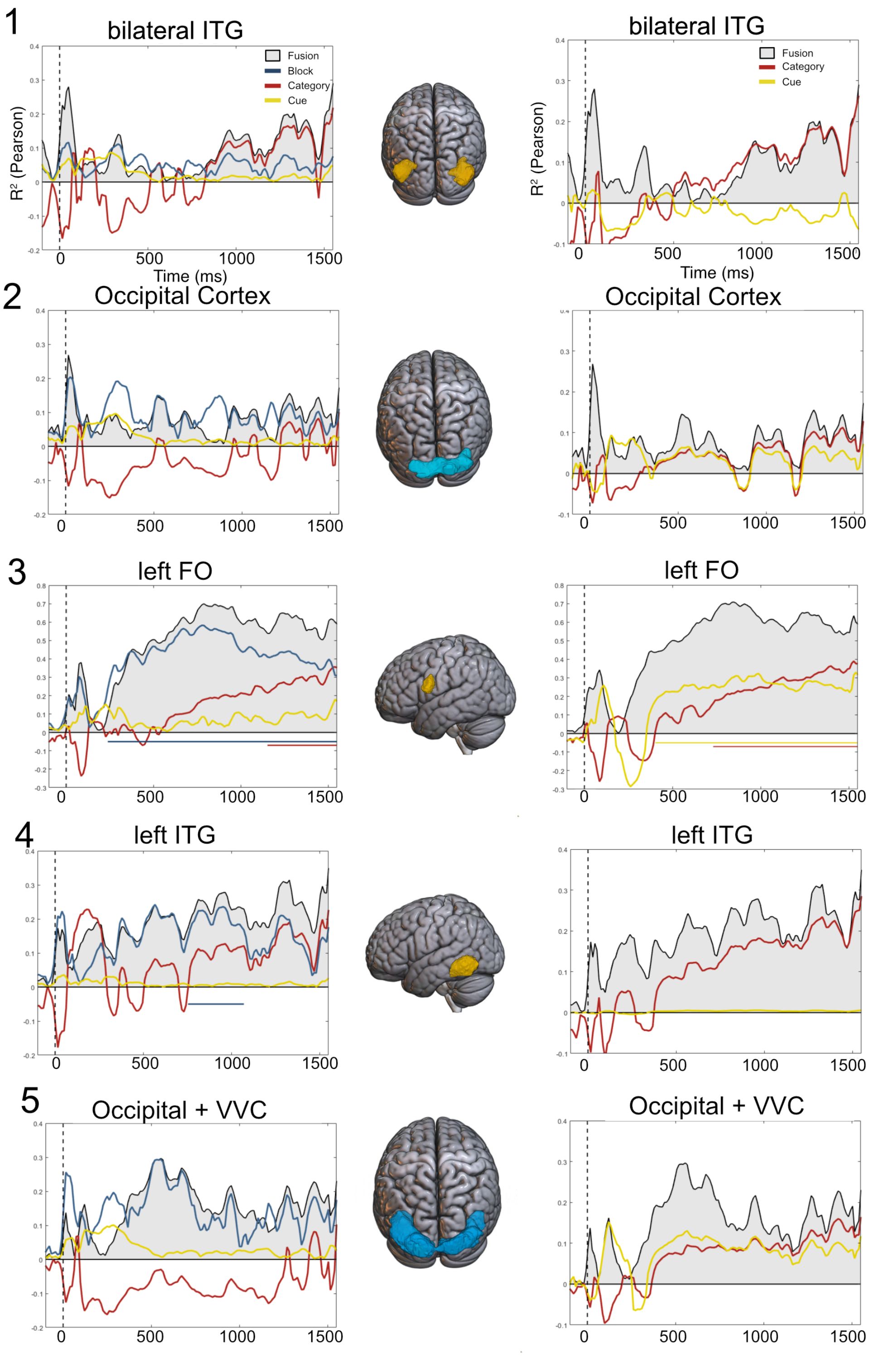

Supplementary Figure 2. Fusion values without applying absolute value. ITG = Inferior Temporal Gyrus; FO = Frontal Operculum.

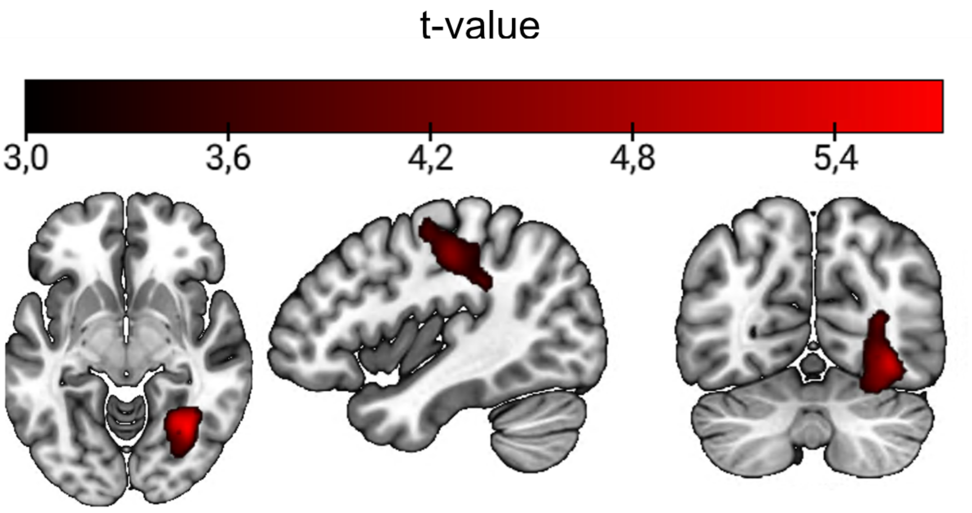

Supplementary Figure 3. Cue-category control analyses. Results only shown for the attention condition, since no significant clusters were found in expectation. The classifier was trained to classify between categories using one pair of cues, and then tested in the other.
